# Questions Asked at the Last Meeting of the State Examining Board, Regular Board of Medical Examiners

**Published:** 1901-05

**Authors:** 


### QUESTIONS ASKED AT THE LAST MEETING OF THE STATE EXAMINING BOARD, REGULAR BOARD OF MEDICAL EXAMINERS.

AUGUSTA, GA., April 2, 1901.

### OBSTETRICS.—DR. J. W. BAILEY.

1. Define placenta previa, giving treatment.
2. Give etiology and treatment of puerperal eclampsia.
3. Give causes of post-partum hemorrhage and the modes of stopping bleeding after labor.
4. How many stages of labor? Describe each.
5. Give mode in which expulsion of the child is effected?

### PHYSIOLOGY.—DR. J. W. BAILEY.

1. State the changes which occur in the blood and air respectively as a result of respiration.
2. State the origin, function and distribution of the fifth pair of cranial nerves.
3. Name the secretions, with the organs engaged in their elaboration; (*b*) the excretions, with the organs by which they are eliminated.
4. Name the great divisions of the nervous system with the chief characteristics and functions of each system.
5. Give the proportion of blood to the weight of the human body.

### CHEMISTRY.—DR. J. B. S. HOLMES.

1. Give two tests for albumin in urine.
2. What is the chemical composition of milk?
3. Give tests for morphia and strychnia.
4. Give the sources and properties of chlorine and name several compounds used in medicine.
5. Give some of the salts of mercury that are used in medicine. Give tests for the mercuric salts.

### GYNECOLOGY.—DR. J. B. S. HOLMES.

1. Describe the method of removing the uterus by both the abdominal and vaginal routes.

2. What do you understand by Alexander's operation? State indications for and methods of performing it.

3. Describe briefly a perineorrhaphy and a trachelorrhaphy.

4. What is the most frequent cause of metrorrhagia after the menopause and how would you treat it?

5. What is pudendal hernia and how would you treat it?

MATERIA MEDICA AND THERAPEUTICS.—DR. E. A. JELKS.

1. Give the action of an irritant on the blood-vessels of the skin and subjacent viscera; cause of such action; name three, one each from the mineral, vegetable and animal kingdom.

2. Give the action of strychnia, therapeutics and dose. Why should the dose not be too frequently repeated? Differentiate poison by strychnia and tetanus.

3. Define sialagogue, galactagogue and cholagogue; give example of each.

4. Write a prescription for chronic malaria, containing quinine, arsenic, strychnia and reduced iron, with extract of gentian as an excipient.

5. Difference in action between digitalis and strophanthus? When is strophanthus preferable to digitalis? Dose of each.

6. Give action of bromides upon the nervous system and sexual organs; therapeutics of bromides. Name two or three preparations with doses.

7. Diagnose a case of arsenic poisoning. Treatment of same?

SURGERY.—DR. F. M. RIDLEY.

1. What are the principles which should govern amputation in general? Describe concisely the essentials for successful amputation through middle of the leg. Name tissues, arteries, muscles divided.

2. Under what circumstances would you delay an operation in acute appendicitis and when would you recommend immediate operation in such cases. Name conditions for which movable kidney may be mistaken. Name and explain symptoms common to both.

3. How would you treat a case of secondary syphilis complicated by gastro-intestinal catarrh and extensive chronic eczema?

How would you treat a case of chronic cystitis in a man forty years old with slight stricture in anterior urethra and an enlarged congested prostate?

4. What is the point of election for ligations of femoral artery in case of popliteal aneurism? Describe the operation. When would ligation in Hunter's canal be indicated?

5. Describe operation for strangulated femoral hernia. Describe an operation for the radical cure of oblique inguinal hernia, naming coverings from without inward.

ANATOMY.—DR. F. M. RIDLEY.

1. Name bones of lower extremity. Name sesamoid bones. What is the composition of bones? Give articulations of ethmoid bone.

2. What is an artery? Name branches of abdominal aorta. Name branches of external carotid. Describe and give relations of arch of aorta.

3. Give origin of pectoralis, major and minor muscles. What muscles does the quadriceps extensor femoris include? Give origin and insertion of the biceps. Give origin and insertion of the biceps. Give origin and insertion of gastrocnemius.

4. Name membranes of the brain. How is the brain divided? Name principal convolutions of cerebrum. Where is fissure of Rolando? Name nerves of special sense.

5. Describe formation and location and distribution of solar plexus.

PRACTICE.—A. A. SMITH, M.D.

1. (*a*) Give two most common varieties of headache; (*b*) causes and appropriate treatment for each.

2. Give prominent symptoms of la grippe and treatment for same.

3. Describe briefly symptoms in angina pectoris and give treatment for same.

4. Give differential diagnostic symptoms in pneumonia and pleuritis.

5. Describe symptoms and give treatment in cholora infantum.

6. Give symptoms in acute laryngitis and treatment for same.

7. Give differential diagnostic symptoms in acute tonsillitis and diphtheria.

8. How would you treat delirium tremens?

9. Describe symptoms and give treatment for gastralgia.

10. Give differential diagnostic symptoms in renal and biliary calculi.

